# Porous Gold Films—A Short Review on Recent Progress

**DOI:** 10.3390/ma7053834

**Published:** 2014-05-14

**Authors:** Renyun Zhang, Håkan Olin

**Affiliations:** Department of Natural Sciences, Mid Sweden University, SE-85170 Sundsvall, Sweden; E-Mail: hakan.olin@miun.se

**Keywords:** porous, gold film, synthesis, characterization, applications

## Abstract

Porous gold films have attracted increasing interest over the last ten years due to the unique properties of high specific surface area and electrical conductivity combined with chemical stability and ability to alter the surface chemistry. Several methods have been developed to synthesize porous gold films such as de-alloying, templating, electrochemical, and self-assembling. These porous gold films are used in diverse fields, for example, as electrochemical and Raman sensors or for chemical catalysis. Here, we provide a short review on the progress of porous gold films over the past ten years, including the synthesis and applications of such films.

## 1. Introduction

Porous materials are used in a diverse set of applications such as metal oxides for electrocatalytic processes or active carbon or graphene for supercapacitors. Porous metals are used due to their catalytic activity and high electrical conductivity with applications in, for example, transistors [1] solar cells [2] and electrochemical sensors [3]. Among the metallic porous films, gold film is of particular interest due to its chemical stability and unique surface chemistry. Gold porous films have seen strongly increasing attention over the last decade with a four-fold increase in the number of publications (as searched in the Web of Science database) from a rather modest level before the year 2000 as described in a previous review [4].

This short review provides a brief overview of porous gold films over the last ten years covering the synthesis of such films, as well as their properties and applications. We hope this review will contribute as a survey of general knowledge of the recent progress on porous gold films.

## 2. Short Story on Earlier Studies

Gold has a long history during the development of human societies and has inspired mankind throughout the ages [5]. Nano-sized gold materials, and in particular nanoparticles have been studied intensely during the last twenty years due to the great application potential in many areas ranging from basic science to industrial use. The main body of work on nanoporous gold materials is, however, more recent and has occurred during the last ten years. More attention on synthetic methods development is found before 2007 [6–16] while more application directed studies have been performed more recently [17–20]. The methods for synthesizing porous gold films include de-alloying [7,10,14], electrochemical deposition [9,21,22], templated electrochemical deposition [6,23], self-assembly [24–27], sputter [28,29], and spray deposition [30]. Spray deposition is a rather new method for deposition of gold film from a gold nanoparticle solution, which is not reviewed as an individual section. Two main applications of porous gold films are as electrodes for chemical sensing [17,23,31–35] and electrochemical catalysis [20,22,36]. However, porous gold films are also of value in other applications, for example, it has been shown that porous gold film could increase the energy conversion rate in a dye sensitized solar cell (DSSC) [37]. More details about the synthetic methods and applications are discussed below.

## 3. Synthesis

There are several methods for fabricating porous gold films, however, we classified them into the following general categories: de-alloying, electrochemical deposition, templated electrochemical deposition, self-assembly, sputter, and spray.

### 3.1. De-Alloying

**Figure 1.**
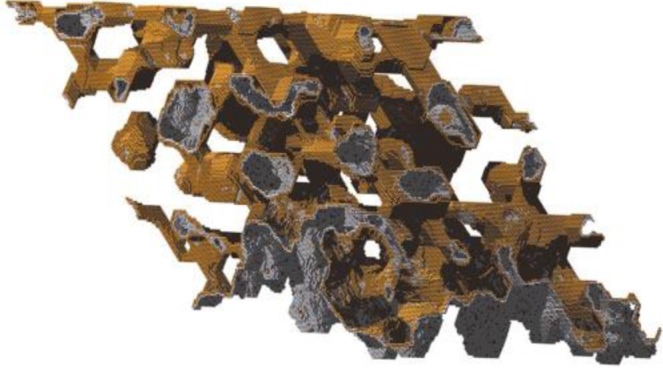
Simulated nanoporous gold. The simulation model was as follows: a bond-breaking model was used for diffusion; atoms with *N* nearest neighbors diffused with rate $k_{N}=v_{D}\text{exp}(-(N_{\epsilon }/k_{B}T)$, where e is a bond energy and *v_D_*_=__10_^13^_s_^−1^. Dissolution rates were consistent with the Butler-Volmer (BV) equation in the high-driving-force Tafel regime; the dissolution rate *k_E,N_* for a silver atom with *N* nearest neighbors was written as $k_{E,N}=v_{E}\text{exp}[-(N_{\epsilon }-\phi /k_{B}T)$, where *v_E_* = 10^4^ s^−1^ is an attempt frequency determined by the exchange-current density in the BV equation and φ is the over-potential. For the Figure, φ = 1.75 eV, ϵ/k*_B_*T = 5.51. Reprinted with permission from [39]. Copyright 2001, Nature Publishing Group.

De-alloying is a corrosive process of alloy components [38] by selective dissolution of the most active elements in the alloy [39]. Eriebacher *et al.* [39] proposed a continuum model (Figure 1) of the de-alloying process of a gold-silver (Au-Ag) alloy. They pointed out that the nanoporosity in alloying metals is due to an intrinsic dynamical pattern formation process, where the pores are formed because of the chemical driven aggregation of gold atoms by a phase separation process [39].

The de-alloying process could be done through two different routes: electrochemical de-alloying and chemical de-alloying. In the electrochemical de-alloying process [40], the electrochemical activities of the metals in the alloy determine the order of dissolution. Gold is at the bottom of the series of metal electrochemical activity, which means it is the metal element that is hardest to dissolve electrochemically. Theoretically, porous gold can be de-alloyed from any gold containing alloys. However, this de-alloying method is not widely used now but examples can still be found on Au-Ag [39] and Au-Zn alloy, [15,41,42] and Au-Li [43]. The porous structures of such nano porous gold films can be tuned by changing the ratio of the alloying metals [40]. Another de-alloying method, chemical de-alloying, also called chemical etching, is based on the chemical activity of metals. Gold is a chemical stable metal and acids or bases can be used to dissolve other metal components, such as silver (Ag), [14,16,18,36,44–47] aluminum (Al), [48,49] and copper (Cu) [50,51]. Recently, polymerized particle-stabilized emulsion gels (bijel) have also been used for templating a porous Au-Ag alloy structure that can be further chemical de-alloyed to a porous gold structure [52].

#### 3.1.1. Electrochemical De-Alloying

**Figure 2.**
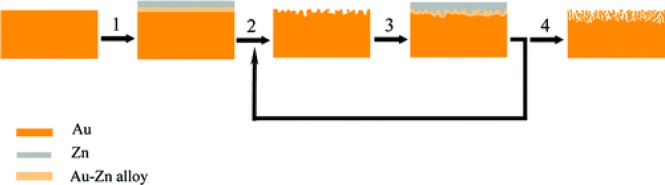
Schematic illustration of the formation of nanoporous gold film electrode by a multicyclic electrochemical alloying/de-alloying method. Step 1, electrodeposition of Zn and formation of Au-Zn alloy; step 2, electrochemical dealloying; step 3, electrodeposition of Zn and formation of Au-Zn alloy again; step 4, formation of nanoporous gold film after multicyclic alloying/de-alloying. Reprinted with permission from [15]. Copyright 2007, American Chemical Society.

Fabrication of porous gold film using electrochemical de-alloying is a two-step method, with one step of electrochemical alloying and the other step of electrochemical de-alloying process. An alloying layer of Au-Ag or Au-Zn is first produced on a pure gold electrode through an electrochemical process, for example, voltammogram scan from −0.72 to 1.88 V (*vs.* Zn, Zn as reference electrode) will create a layer of Au-Zn alloy on a gold electrode using ZnCl_2_/benzyl alcohol as electrolyte [15,41]. However, the voltage range of voltammogram scans is highly depending on the electrolyte. If one chooses ZnCl_2_/1-ethyl-3-methylimidazolium as electrolyte, the range is from −0.2 to 1.0 V (*vs.* Ag/AgCl) [42]. By voltammogram scanning of such an alloyed electrode from positive to negative voltage, the alloying layer will be destroyed where the Zn or Ag in the alloy will be dissolved, leaving a porous gold layer. By repeating such a process (Figure 2) using the cyclic voltammogram method, porous gold layers with different thicknesses can be created [15,41]. These methods alloy metals to the gold electrode, however, the alloying can also be prepare by introducing gold to other metal substrates such as copper [23,53]. Beside the alloying/de-alloying routine, there are methods that only use the de-alloying step by removing metal from a premade alloy such as Cu_3_Au through electrochemical decomposition [47].

#### 3.1.2. Chemical De-Alloying

Chemical de-alloying/etching [54] is done by dissolving metal elements such as Ag [4,7,9,18] Sn [55] and Cu [56] in the alloy except for gold. Based on the chemical activity of the elements in the alloys, different reagents are used such as nitric acid [4,14,18,20,44,50,57,58] hydrochloric acid, [50] or NaOH [10,49,59,60]. To dissolve Ag from Au-Ag alloys, nitric acid [4,7,9,18,20,58,61,62] is commonly used since Ag is a relatively low chemically active metal. Beside nitric acid, there are also examples using the reaction between Ag and AuCl_4_^−^ to replace the Ag with Au in a film of mixed Ag and Au nanoparticles, resulting in a nanoporous gold film [9,63]. For other metals with higher activity such as Cu, there are more choices of acids like nitric acid and hydrochloric acid [50]. For Au-Al alloy, usually in the form AuAl_2_, NaOH is used to dissolve the Al (which is also a standard method to dissolve metal Al) [10,49,59,60].

To create more complicated structure, such as hierarchical structure, one can combine chemical de-alloying with polymer templating. Lee and co-workers [52] reported a universal platform for synthesizing monolithic porous gold using polymerized bijel as template, creating hierarchical bicontinuous morphology and combined macro- and mesoporosity.

### 3.2. Electrochemical Deposition

**Figure 3.**
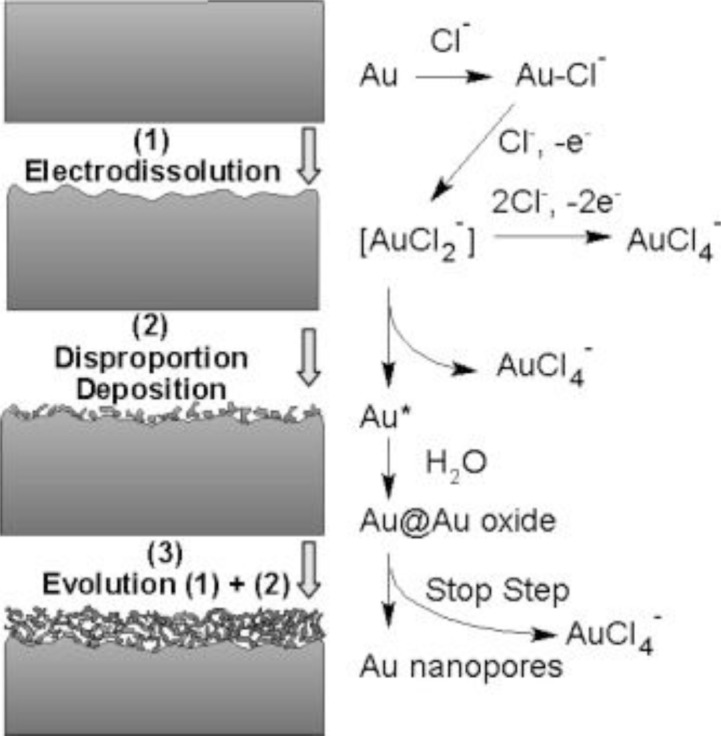
Schematic illustration of the fabrication principle of nanoporous gold films. Reprinted with permission from [64]. Copyright 2008, Elsevier.

In contrast to the electrochemical de-alloying methods mentioned above, electrochemical deposition does not include the alloying and de-alloying processes. There are different approaches to achieve electrochemical depositions of porous gold. Deng and co-workers developed a facile method in an electrochemical cell based on the electrochemical reaction between gold and HCl, where a process of electrodissolution-disproportion-deposition is involved (see Figure 3) [64]. A gold substrate undergoes first active electrodissolution under a diffusion control of HCl, forming AuCl_2_^−^; then the AuCl_2_^−^ immediately disproportionates in the Au atoms; and as the last step, the Au atoms aggregate and deposit on the gold substrate, leading to a porous gold film [54,61,65].

Another widely used method to deposit porous gold film is to reduce Au ions from HAuCl_4_ [17,21,33,35,66] Gold nanoparticles are reduced from HAuCl_4_ by adding a constant potential [67,68] to the substrates which is dependent on the material, e.g., 0.28 V (*vs.* SCE, saturated calomel electrode) on a indium tin oxide (ITO) electrode [33], −0.5 V (*vs.* Ag/AgCl) on a glassy carbon electrode (GCE) in the presence of lead acetate [17], 0.5 V (*vs.* SCE) on a GCE without help of other ligands [66], −0.1 V (*vs.* Pt) on a gold electrode in the presence of lead(IV) acetate [21], and 0.5 V (*vs.* SCE [31,69] Ag/AgCl^35^) on a gold electrode in the presence of HClO_4_. After the formation of nanoparticles, they then aggregate into a porous gold film on these substrates. These methods are usually combined with a templating procedure that is discussed below.

In a third method, electrochemical scans are used to create layers of anodized gold followed by chemical reduction. The anodization procedure is usually done in phosphate buffers (PBS), but with different electrochemical techniques. It can be achieved by applying a potential to the electrode, for example 10 V (*vs.* SCE) for 3 min on a gold electrode in 0.1M PBS (pH 7.4) [13], 10 V (*vs.* SCE) for 5 min in 0.1 M PBS (pH 7.0) [70], 5 V (*vs.* Ag/AgCl^19^ SCE^22^) for 3 min in 0.15 M (pH 7.4), or by applying a step potential from open circuit to 4 V (*vs.* Ag/AgCl) [35,71,72] or 5 V (*vs.* Ag/AgCl) [73] in a 0.2M PBS (pH 7.4), or by applying a square wave potential pulse between 0.8 and −0.5 V (*vs.* SMSE, saturated mercurous sulfate electrode) at 50 Hz in a NaOH solution [74]. The oxidation process creates a salmon pink layer of anodized gold, and the color turns to black after the reduction, indicating the formation of nanoporous gold films [13].

In addition to the above-mentioned methods, there are also other electrochemical methods to obtain porous gold films. For example, a porous gold film can be created by a gold-plating process [75].

### 3.3. Templated Electrochemical Deposition

**Figure 4.**
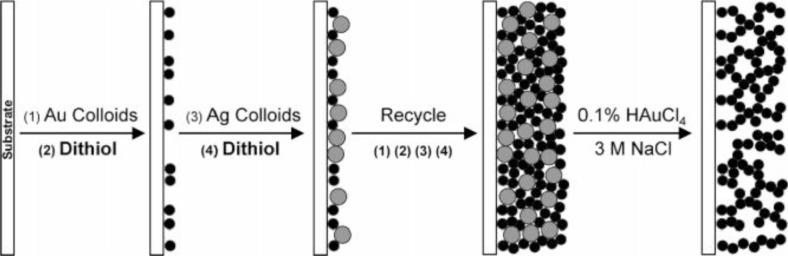
Schematic illustration of the preparation of multilayers of colloidal Au/Ag and the subsequent removal of colloidal Ag templates to form porous Au films. Reprinted with permission from [9]. Copyright 2005, American Chemical Society.

Templating is a simple method that can combing with other techniques like flow-stream technique [76] sputter deposition (Section 3.5) and electrochemical methods (Section 3.3) to create nanoporous gold films (Figure 4) [9]. This section mainly focuses on the combination of template and electrochemistry. The main steps in the procedure are first a preparation of template, followed by deposition of gold, and then the removal of the template [77,78]. The templates could be a layer of assembled particles [31,78], biologically made templates [8] or ion etched substrates [6]. Polystyrene latex [33,78] and silica particles [31,34] are commonly used to make assembled particles as templates. Gold is then electrochemically deposited on the templates in solution [31,33,34,70,78]. These methods for depositing gold are electrochemical ones, where Au is electrochemically reduced from HAuCl_4_ [17,21,33,35,66]. Details of these electrochemical processes are described in Section 3.2. Beside the above mentioned templating methods, there is another method to fabricate porous gold film in liquid phase using the droplet condensation of water, where the condensed water droplets create the pores and gold nanoparticles are deposited at the spaces among the droplets [11].

### 3.4. Self-Assembly

**Figure 5.**
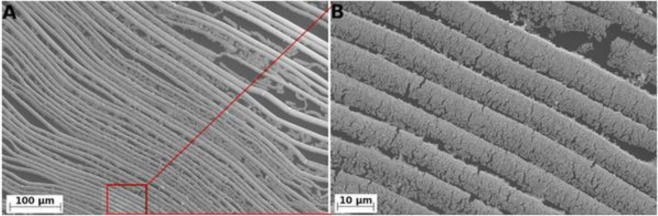
SEM images of gold nanobelts at low (**A**) and high (**B**) magnification. Reprinted with permission from [25]. Copyright 2012, PLOS ONA.

Self-assembly does not rely on external assistance, as in the templating methods described above, but the self-assembly process of the gold nanoparticles leads to the porous structure directly. The formation of porous structure is based on the aggregation and coalescence of gold nanoparticles. Renyun Zhang and co-workers developed an evaporation induced self-assembly method to produce porous gold films from colloidal gold solution [24–27]. The fabrication of such porous gold films could be done by placing the gold nanoparticle solution in an ambient condition and letting water evaporate. The gold nanoparticles are first concentrated at the capillary meniscus and then coalesce [26] into nanowire like structures. The growth is then extended to the surface of the colloidal gold solution, leading to a porous gold film [24]. By controlling the evaporation conditions, different porous morphologies are achieved [27], even highly anisotropic structures (Figure 5) [25].

### 3.5. Sputter Deposition

Sputter deposition [28,29] of porous gold film can be a sole templating process [79] or a combination of templating and de-alloying [80]. The templates for gold deposition can be created by UV light [81] or electrochemical etching [79]. By photolithography, patterned templates for deposition can be created [81] where the patterning is usually defined by an optical mask. Wi and co-workers [80] created a more complicated porous gold structure, where they created pores in a template porous gold film by a de-alloying technique. Briefly, a porous template is first created by lithography, then Au-Cu alloy is sputtered on such a template then followed by de-alloying. After sputtering, porous Au-Cu alloy film is formed, however, smaller pores are later created when Cu is dissolved. Such smaller pores can be used as hot spots for Raman scattering.

## 4. Properties of Porous Gold Film

### 4.1. Optical

Maaroof and co-workers have measured the optical properties of mesoporous gold film using spectrophotometry and ellipsometry, and they pointed out that such gold films exhibit unique dispersion in their optical response across all NIR wavelengths [10]. The optical constant n* (refractive index) displays non-metallic characteristics, while k* (extinction coefficient) shows weak metallic character [10]. Later, they modulated the spectral response of porous gold film using a homogeneous Lorentz-Drude (L-D) model, and showed that the optical properties of nanoporous gold films are dependent on the occupancies of voids [48,59]. Dixon *et al.* [14] showed that the spectral features are depending on the thickness of the porous gold films, where a thin porous gold film is similar to bulk gold, while thicker films have novel properties.

### 4.2. Electrical

A porous gold film can be seen as a network of gold wires with non-uniform diameters. Electrically, it is a conductive film, however the conductivity is influenced by the morphology of the film. For example, Zhang and coworkers [24–26] demonstrated that the sheet resistance of porous gold films, self-assembled at different temperature, could have a sheet resistance from 25 to 120 Ohm/sq. The electrical conductivities of porous gold films can be measured using a configuration similar to common electrical devices, however, it can also be obtained by using an open-terminal method at microwave frequencies [82]. Using this method, the alternating current (ac) conductivity of gold films can be measured, which indicates that the ac conductivity of a porous gold film could be higher than the direct current (dc) conductivity in some case due to the surface state of the film [82].

### 4.3. Surface Area

Porous gold has a specific surface area (SSA) about 10 m^2^/g, when the pore sizes are around 30–40 nm [5]. Smaller pore size gives higher SSA, for example, Qin and co-workers produced a mesoporous gold sponge with SSA of 11.9 m^2^/g with pore sizes of 5–30 nm. Fujita and co-workers reported similar SSA of 12 m^2^/g with a pore size of ~7.5 nm [83]. If the pore size increases to 1 μM, such as in the case of templating by polystyrene beads and then de-alloyed with Ag, the SSA is 1.48 m^2^/g with ligament spacings of 10–100 nm [16].

Another parameter that is used to define the porosity of a porous gold film is the roughness parameter (R), which is the ratio of the real surface area to the geometric surface area [23]. The roughness factor could go over 1000 if the porous film was electrochemically deposited on gold disk in an electrolyte of mixed NaBH_4_, KCl and mercaptoundeconic acid (MUA) [84]. Lizhi Zhang’s group reported a roughness factor of 560, where the porous films are produced with pore sizes of 30–200 nm [15] or 60–100 nm [46] by electrochemically de-alloying Au-Zn alloy. If the porous gold film is prepared from the electrochemical interaction between Au and Cl^−^, the roughness factor could reach 218 [61]. However, the roughness parameter of most of the studies is between ten to one hundred. The values vary due to different film preparing methods and pore sizes [7,15,18,22,33,34,53,64,65,69,71–74,78,85].

## 5. Applications

Porous gold films are ideal electrodes for the development of chemical/biological sensors [58]. The most common sensor type based on porous gold is the electrochemical one due to the high conductivity, large surface area, chemical stability, and ease of modification. Porous gold films are also an excellent substrate for Raman scattering with applications as Raman sensors [86]. The transparency and high conductivity of porous gold films can also be applied in electronic devices like dye-sensitized solar cells [37]. The elasticity of porous gold films can also be employed for making electronic devices like force sensors [25].

### 5.1. Electrochemical Sensors

#### 5.1.1. Glucose Sensing

Glucose is an important indicator for diagnosing diabetes, giving glucose sensors an important role in the monitoring and control of diabetes. Electrochemical glucose sensors can be categorized into two types: enzyme-free [87,88] and enzyme-participated [89]. However, porous gold film based electrochemical glucose sensors are mainly enzyme-free [13,23,33,61,66,90] and due to the high surface area, these sensors have a high sensitivity. Enzyme-fee operation gives these sensors several advantages such as easy fabrication, high stability, and simplified storage.

**Figure 6.**
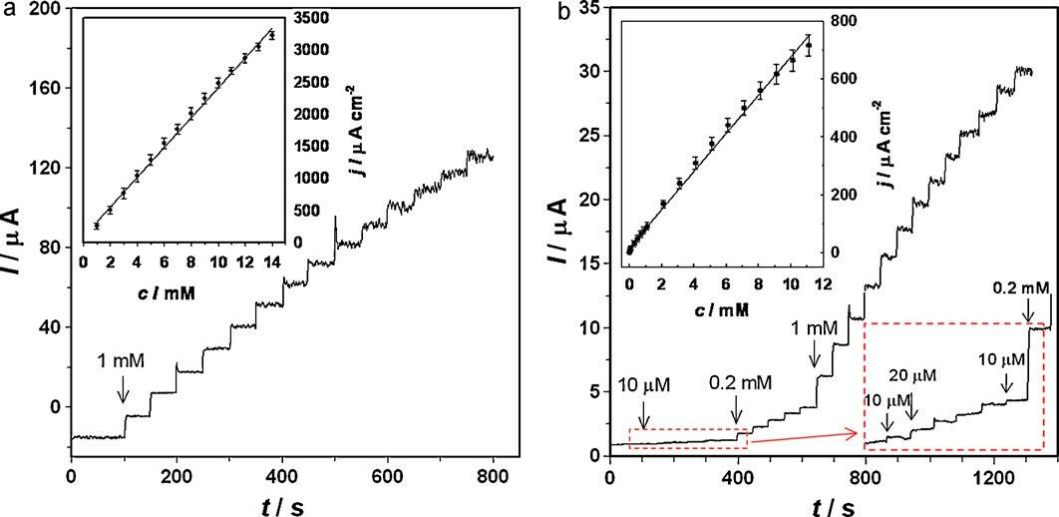
Amperometric responses of the NPGF (Nano porous gold film) electrode to successive addition of glucose in a continuously stirred solution of 0.1 M PBS (Phosphate buffer) (pH 7.4) containing (**a**) 0.1 M Na_2_SO_4_ at −0.15 V (Reference electrode: SCE, saturated calomel electrode) or (**b**) 0.1 M NaCl at 0.2 V (Reference electrode: SCE). The upper left insets shows the corresponding calibration curves. The error bars indicate the standard deviation of triplicate determinations. The lower right inset in (**b**) is a local enlargement marked with a dashed box, showing the lowest detectable concentration of 10 μM. Reproduced with permission from [61]. Copyright 2011, Elsevier.

The sensitivity of such enzyme-free glucose sensors [91] based on porous gold films depends on the preparation methods of the porous films and the supporting electrode. A porous gold film made on a gold electrode by first anodizing the gold electrode and then reducing with β-D-glucose is able to detect 0.75 μM glucose at a potential of 0.3 V (*vs.* SCE) in pH 7.4 PBS solution [13] while a porous gold film on a gold electrode made with galvanic replacement reaction shows a detection limit of 5 μM at a potential of 0.35 V (*vs.* SCE) [23,55]. The detection range could also vary, with a range of 0.75–57.5 mM for the first example and 2–10 mM for the second one. Xia and co-workers [61] also reported an enzyme-free glucose sensor based on the porous gold film made by the electrodissolution-disproportion-deposition process [64], with a detection limit of 8.7 μM and a linear range of 10–11 mM (Figure 6).

#### 5.1.2. Protein Sensing

Protein sensors may detect protein directly, indirectly, or through immunoreactions. The proteins suitable for direct detections are usually heme-containing redox proteins, such as cytochrome *c*^53^ or hemoglobin [31,92]. Li and co-workers [53] reported an electrochemical method for direct detecting cytochrome *c* on a porous gold film. The connection of 11-mercaptoundecanoic acid between gold and cytochrome *c* was used to form a monolayer of cytochrome *c* on a porous film, leading to a high heterogeneous electron transfer rate with a constant (k_s_) of 1.73 s^−1^ that is higher than on other kinds of gold electrodes. One of the advantages of such porous gold films is the high surface area, leading to high degree of protein adsorption. Wang and co-workers [31] reported a ten times higher hemoglobin adsorption on a three-dimensionally ordered macroporous gold film than on flat gold electrodes. Similarly, 3.3 times higher adsorption of protein SbpA from *Bacillus sphaericus* CCM 2177 has been found on porous gold [85]. Beside heme-containing redox proteins, there are also other proteins such as horseradish peroxidase that could be detected on nanoporous gold film, however, functionalization of such porous gold film might needed [93].

An indirect electrochemical protein sensor utilizes another electrochemical indicator to show the presence of the target protein, where the protein triggers the electrochemical signal of the indicator. An example of such a sensor is the aptasensor that is used to detect adenosine triphosphate (ATP), which is a vital substrate in living cells as the mediator of energy exchange. To detect ATP electrochemically, one needs molecular reporters such as 3,4-diaminobenzoic acid (DABA) [35] or quantum dots [69] to indicate the presence of ATP. However, with the help of porous gold film, ultrahigh detection limit can be achieved at 100 nM using DABA as reporter [35] and 0.01 nM with the help of quantum dots [69].

**Figure 7.**
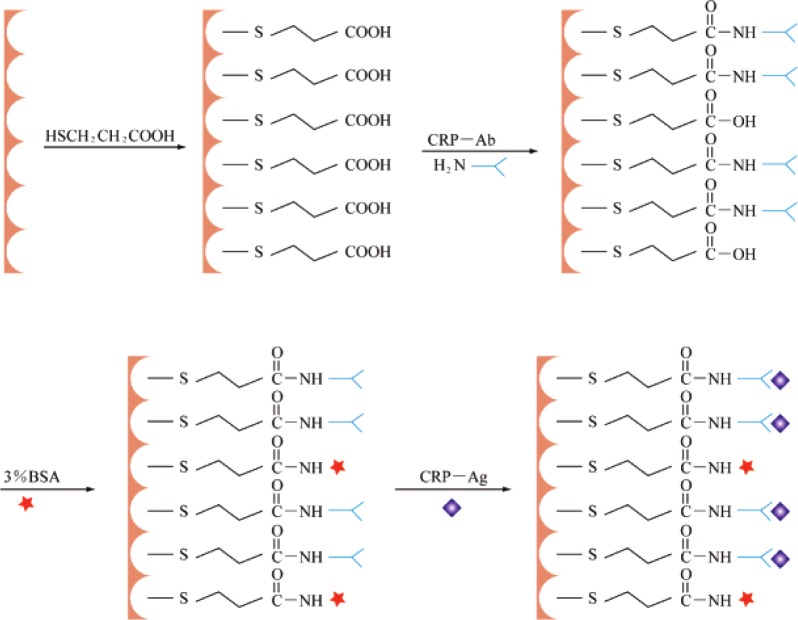
Schematic illustration of the stepwise immunosensor fabrication process. Reprinted with permission from [34]. Copyright 2008, American Chemical Society.

An electrochemical immunosensor is a device to detect the interaction between antigen and antibody. Immunosensors built on porous gold films can cover a large range of immunoreactions by functionalizing the porous gold films with different adsorbed proteins. Antigens like carcinoembryonic antigen (CEA) [94], IgG [17], C-reactive protein (CRP) [34], prostate specific antigen (PSA) [44], *etc.* have been used in such sensors. Figure 7 shows a schematic drawing of the process for fabricating an immunosensor [34]. Ultra-sensitive detections of immunoreactions are achieved using this kind of sensor, for example, 0.06 ng/mL for CEA [94], 0.009 ng/mL for IgG [17], 0.1 ng/mL for CRP [34], and 3 pg/mL for PSA [44], can be detected on different porous gold film based immunosensors.

#### 5.1.3. DNA Sensing

Electrochemical DNA sensing detects the signal changes of indicators before and after DNA hybridization [95,96]. The contributions of porous gold films in this area are the high specific surface area and easily modifiable surface. For example, thiol or amino group labeled DNA strands can be strongly bonded to a gold surface. Using the amplification effect of the nanoporous gold combining with gold nanoparticles [97]. Hu and co-workers [98] fabricated a DNA sensor with a detection limit of 28 aM. Such a type of DNA sensor can be combined with luminescence technique [99] to detect target DNA sequence through an electrochemiluminescence signal, such as CdTe quantum dots [100]. These kinds of DNA sensors can not only be used to detect DNA sequences, but also can be extended for the detection of bacteria. Li and co-workers reported a study of a nanoporous gold-based electrochemical DNA biosensor to detect *Escherichia coli* (*E. coli*) with a detection limit of 50 cfu/mL [19].

#### 5.1.4. Molecular Sensing

Porous gold film electrodes are ideal platforms for determining some small molecules, because these molecules are electroactive and have a low electron transfer rate and/or adsorb on gold [58]. Different kinds of molecules have been found to be electroacitve on porous gold film electrodes such as dopamine, [15,101], ascorbic acid [73], hydrogen peroxide [102,103], and glucose [13,23,33]. In this review, glucose sensors are described separately above because of the special interest they have received.

#### 5.1.5. Mercury Sensing

Besides biological electrochemical sensors, porous gold films can also be used for detecting metal ions, such as mercury. Mercury is a harmful heavy metal, and the commonly used methods for detecting mercury require a large surface area electrode [104] leading to the use of porous gold films. Zhang and co-workers reported an optical sensor for detecting mercury ions based on an aptamer modified nanoporous gold film, where the detection sensitivity can be improved to 1 pM [105]. Oh and co-workers fabricated an electrochemical mercury sensor based on 1,6-hexanedithiol modified porous gold film, giving a linear signal range of 0–30 μM [106].

### 5.2. Raman Sensors

**Figure 8.**
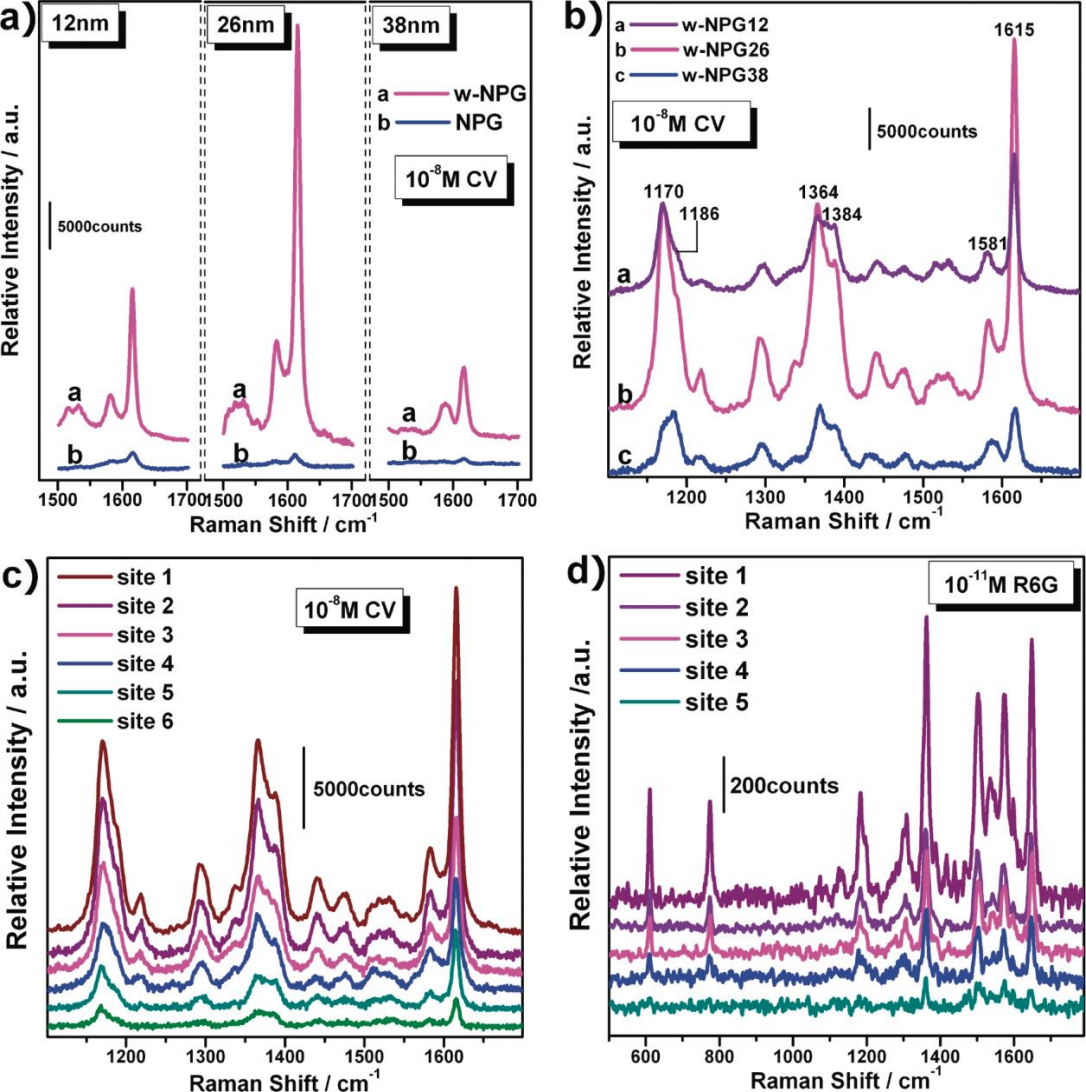
Surface enhanced Raman scattering (SERS) spectra of crystal violet (CV) and rhodamine 6G (R6G) molecules on wrinkled nanoporous gold (NPG) films. (**a**) Comparison of SERS intensity between wrinkled and as-prepared NPGs with different nanopore sizes; (**b**) SERS spectra from wrinkled NPG with different nanopore sizes of 12 nm(w-NPG12), 26 nm (w-NPG26), and 38 nm (w-NPG38); (**c**) variation of SERS spectra of CV on w-NPG26 at different sites along a wrinkle ridge; (**d**) variation of SERS spectra of R6G on w-NPG26 at different sites along a wrinkle ridge. The excitation wavelength is 632.8 nm for CV and 514.5 nm for R6G. Reprinted with permission from [86]. Copyright 2011, American Chemical Society.

Porous gold films have been demonstrated to be excellent substrates for surface enhanced Raman scattering (SERS) with, for example, long SERS stability as in electrochemically fabricated nanoporous gold film [65]. However, the enhancement of Raman scattering is more important, and the enhancement of SERS by porous gold is linearly proportional to the curvatures of the nanopores [83,107]. Gold film with smaller nanopores shows a higher SERS signal as demonstrated by Qian and co-workers [107] where they tuned the nanopore size from ~33 nm down to ~5 nm. Using nanoporous gold, they achieved a detection limit of ~5 × 10^−10^ M for rhodamine 6G (R6G) that is much better than on a flat gold substrate [108]. Huang and co-workers found that the SERS enhancement of three-dimensional hierarchical porous gold films can be further improved and repeatedly recovered by treating the films with an “oxidation-dissolution” pretreatment process in NaOH and HCl sequentially [74]. Recently, Ling Zhang and co-workers [86] achieved 100 times higher enhancement for detecting crystal violet (CV) and rhodamine 6G by wrinkling nanoporous gold films (Figure 8). Recently, Osinkina and co-workers reported a ~10^5^ enhancement of SERS on a monolayer gold nanostar array over an area of several hundreds of square micrometers [109].

The examples above show great progress in SERS determination. However, the enhancement factors of those porous gold films produced by de-alloying are influenced by the contamination of alloyed metal. Zhang and co-workers [110] pointed out that Ag residual in porous gold film can improve the SERS effect, and it can be further improved if the residual is homogenized.

### 5.3. Other Applications

**Figure 9.**
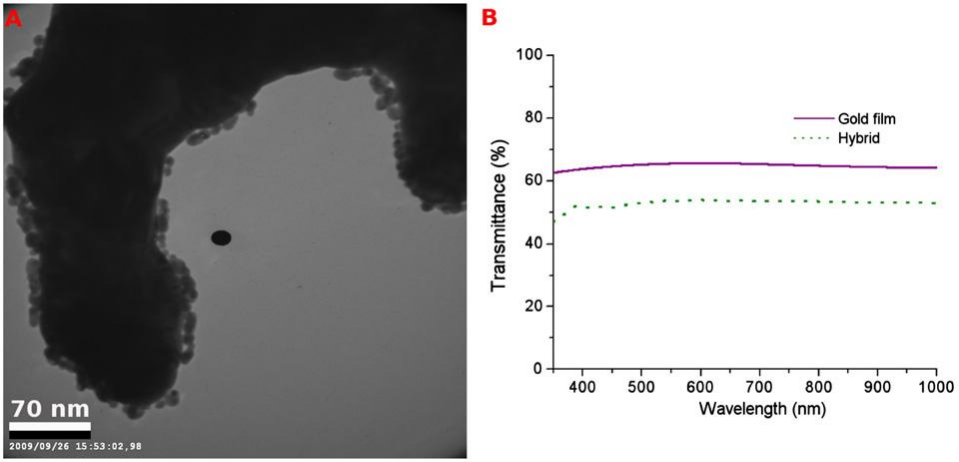
(**A**) TEM image of gold film/gold nanoparticle hybrid; (**B**) the transmittance of gold film and gold film/gold nanoparticle hybrid. Reproduced with permission from [26]. Copyright 2010, Elsevier.

Porous gold films are applied in many other fields, in addition to the applications mentioned above. Hieda and coworkers [7] developed a porous gold electrode based ultrasensitive quartz crystal microbalance (QCM), where the high effective adsorbing surface area of such porous gold electrodes enhanced the sensitivity of the balance by a factor of 40. Using this QCM, a change of 4 × 10^−4^ layer in the coverage of an adsorbed helium film can be detected [7]. Renyun Zhang and co-workers [25] developed a force sensor by using the elastic property of a single layer porous gold film. They also grew patterned ZnO nanowires on patterned single layer porous gold films grown by self-assembly [25]. Moreover, they placed a single layer porous gold film at the bottom of a TiO_2_ layer and used it as a current collector in a dye-sensitized solar cell (DSSC) using the transparency of the porous gold film, and achieving an energy conversion efficiency of 2.8% without any optimizing procedures [37]. Furthermore they proposed an optical biosensor using the transparency change of the porous gold film when extra gold nanoparticles were added to the film (Figure 9) [26].

Electrocatalysis of the oxidation of methanol is another application of porous gold films. Gold electrodes are an effective catalyst for methanol oxidation in alkaline solutions [46]. Compared to flat gold electrodes, porous gold electrodes have a higher electrocatalytic activity due the high surface area. It has also been demonstrated that a porous gold electrode has a higher stability during direct oxidation of methanol [46]. Moreover, the electrocatalytic activity and the stability can be further improved by introducing other metals into the porous film, such as platinum (Pt) [36].

Besides electrocatalysis, nanoporous gold films can also be used as catalysts for selective gas-phase oxidation of methanol at low temperature. The catalytic selectivity can reach 97% at temperatures below 80 °C [111]. Similarly, nanoporous gold films can be used to catalyze the oxidation of CO and Wittstock and co-workers [20,112] reported several works on the catalytic oxidation of CO at 80 °C.

## 6. Summary

We reviewed here the progress of porous gold films over the last ten years, emphasizing the synthetic methods, properties and applications. The reviewed works demonstrated that porous gold films can be prepared by simple methods like de-alloying, templating, and self-assembly and can be utilized in many fields. However, the applications during the last ten years have so far been mainly towards chemical sensing although other applications have been demonstrated.

In our opinion, there are several aspects of porous gold that need further attention:

- Free-standing films of porous gold could be of importance in future applications as transparent flexible electrodes with high electrical conductivity, however, up to now the main body of synthesis of porous gold has been on bulk substrate. Applications include transparent conductive electrodes for solar cells or other electronic applications where these properties are required.
- The optical applications of these porous gold films deserve more attention. The surface chemistry of gold provides an excellent platform to functionalize the surface with different chemicals or bio-matters like DNA and proteins. Combing the transparency of the porous gold film with the opportunities offered by gold surface chemistry could result in the development of high sensitive optical sensors.
- The application of porous gold films in storing energy is another area that could be of importance. Use of the high specific surface area could be applied to supercapacitors when combined with other materials like graphene.

Beside these, there are more aspects that could be studied in the future, such as the application of porous gold films in medical diagnoses. Porous gold films will attract further attention in the future and are an attractive material for both fundamental and applied studies.
